# 

**Published:** 1878-01

**Authors:** 


### CONTENTS.

### COMMUNICATIONS.

The Effects of Anæsthetics upon the Human System, as Evidenced by Spectroscopic Observations.—*By Dr. S. Waterman* ........ 1

Galvanic Action within the Mouth. Its Relation to Dental Surgery. *By Geo. Watt* ........ 23

Conservation of Vital Force.—*By Dr. J. Williams* ........ 33

### EDITORIAL.

A New Dental Journal ........ 40

Nebraska State Dental Society ........ 41

Cyanids of Zinc in Facial Neuralgia ........ 41

Poisoning by Burning Gas ........ 42

Why is Hygiene not Taught in Medical Colleges? ........ 42

Board of Dental Examiners ........ 44

